# 935. Antibiotic Use (AU) Adjustment by Infection-Related Patient Volume Across a Health System

**DOI:** 10.1093/ofid/ofac492.779

**Published:** 2022-12-15

**Authors:** Steven Shaefer Spires, Elizabeth Dodds Ashley, Travis M Jones, April Dyer, Alicia Nelson, Deverick J Anderson, Melissa D Johnson, Christine Zurawski, Todd Parker, Rebekah W Moehring, Moiz Master, Melina Diaz, Oida Corry-Wiggins, Angelina Davis

**Affiliations:** Duke University School of Medicine, Durham, North Carolina; Duke Center for Antimicrobial Stewardship and Infection Prevention, Durham, North Carolina; Duke Center for Antimicrobial Stewardship and Infection Prevention, Durham, North Carolina; Duke Center for Antimicrobial Stewardship and Infection Prevention, Durham, North Carolina; Duke University School of Medicine, Durham, North Carolina; Duke University, durham, North Carolina; Duke University School of Medicine, Durham, North Carolina; Piedmont Health, Atlanta, Georgia; Piedmont Health, Atlanta, Georgia; Duke University, durham, North Carolina; Piedmont Health, Atlanta, Georgia; Piedmont Health, Atlanta, Georgia; Piedmont Health, Atlanta, Georgia; Duke Center for Antimicrobial Stewardship and Infection Prevention, Durham, North Carolina

## Abstract

**Background:**

Benchmarking AU is important to identify opportunities and allocate resources within a health system. Patient level factors such as infection diagnosis codes further refine risk adjustments but have not been more widely adopted because of the burden of accurately collecting and submitting granular data. The goal of this study was to evaluate a novel metric to estimate facility-level infection burden as a potential factor to use in adjustment of AU.

**Figure:**
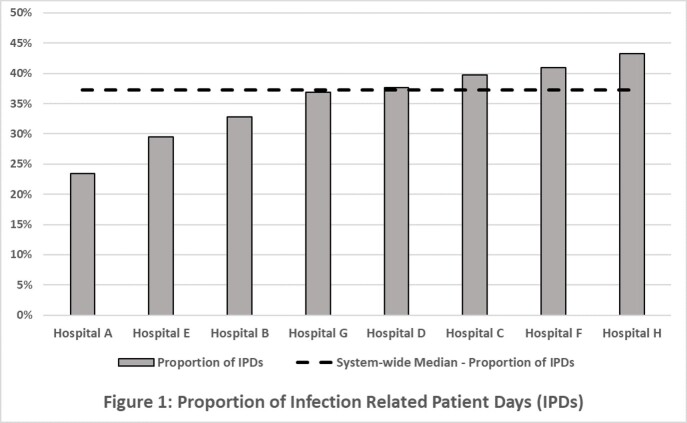

**Figure:**
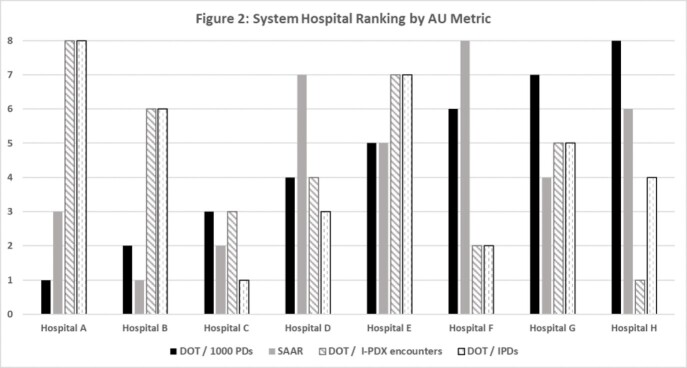

**Methods:**

We conducted a retrospective analysis of hospital administrative data (for calendar year 2020) from 8 hospitals in a single health system using a common electronic health record and coding department. We identified inpatient encounters with an infection-related primary ICD-10 code (I-PDX), based on the health system’s coding department determination and extracted the length of stay (LOS) for each encounter. For any encounter with an I-PDX, the entire LOS was classified as infection-related patient days (IPD). Overall AU in days of therapy (DOT) was adjusted using two novel infection diagnoses denominators. The first was based on proportion of total patient days (PD) attributable to I-PDX encounters (% I-PDX x PD). Since LOS tends to be longer in I-PDX, we also calculated DOT with adjustment for actual extracted IPDs. We then rank ordered study hospitals based on standard DOT / 1,000 PD, NHSN SAAR metrics, and our novel DOT / (% I-PDX x PD) and DOT / 1,000 IPD metrics.

**Results:**

The proportion of I-PDX was highly variable among hospitals, with a system-wide median of 37.27% (range 23.48 - 43.32) (Figure 1). Using DOT / 1,000 patient days for 1 year, Hospital A was the lowest in the system and hospital H was the highest (Figure 2). However, after adjusting for the proportion of patients with I-PDX encounters and IPDs, hospital rank changed considerably, i.e. Hospital H and C respectively ranked lowest and Hospital A was highest.

**Conclusion:**

These novel infection diagnoses PD denominators more closely associated facility level infection burden with AU, for a more refined rank order within the health system. These metrics provide an example of a parsimonious adjustment using patient level data that is already collected at any facility. Next steps might include indirect standardization using PDX categories and other patient level factors readily collected.

**Disclosures:**

**Melissa D. Johnson, PharmD**, Charles River Laboratories: Grant/Research Support|Entasis: Honoraria|Merck: Grant/Research Support|Pfizer: Grant/Research Support|Scynexis: Grant/Research Support|Theratechnologies: Grant/Research Support|UpToDate: Honoraria **Rebekah W. Moehring, MD, MPH, FIDSA, FSHEA**, UpToDate, Inc.: Author Royalties **Angelina Davis, PharmD, M.S.**, Merck & Co.: Honoraria.

